# Alternative Splicing During the *Chlamydomonas* *reinhardtii* Cell Cycle

**DOI:** 10.1534/g3.120.401622

**Published:** 2020-08-18

**Authors:** Manishi Pandey, Gary D. Stormo, Susan K. Dutcher

**Affiliations:** Department of Genetics, Washington University in St. Louis, St. Louis, MO 63105

**Keywords:** Alternative splicing, Chlamydomonas reinhardtii, Diurnal cell cycle

## Abstract

Genome-wide analysis of transcriptome data in *Chlamydomonas reinhardtii* shows periodic patterns in gene expression levels when cultures are grown under alternating light and dark cycles so that G1 of the cell cycle occurs in the light phase and S/M/G0 occurs during the dark phase. However, alternative splicing, a process that enables a greater protein diversity from a limited set of genes, remains largely unexplored by previous transcriptome based studies in *C. reinhardtii*. In this study, we used existing longitudinal RNA-seq data obtained during the light-dark cycle to investigate the changes in the alternative splicing pattern and found that 3277 genes (19.75% of 17,746 genes) undergo alternative splicing. These splicing events include Alternative 5′ (Alt 5′), Alternative 3′ (Alt 3′) and Exon skipping (ES) events that are referred as alternative site selection (ASS) events and Intron retention (IR) events. By clustering analysis, we identified a subset of events (26 ASS events and 10 IR events) that show periodic changes in the splicing pattern during the cell cycle. About two-thirds of these 36 genes either introduce a pre-termination codon (PTC) or introduce insertions or deletions into functional domains of the proteins, which implicate splicing in altering gene function. These findings suggest that alternative splicing is also regulated during the *Chlamydomonas* cell cycle, although not as extensively as changes in gene expression. The longitudinal changes in the alternative splicing pattern during the cell cycle captured by this study provides an important resource to investigate alternative splicing in genes of interest during the cell cycle in *Chlamydomonas reinhardtii* and other eukaryotes.

Alternative splicing (AS) is a ubiquitous process that occurs in most known eukaryotes (Irimia *et al.* 2007). AS facilitates production of multiple protein-coding transcripts from a finite set of genes and offers another level of gene regulation (Lee and Rio 2015). AS events are usually classified into five major categories: Exon skipping (ES), Alternative 5′ (Alt 5′), Alternative 3′ (Alt 3′), Mutually exclusive exons (ME) and Intron Retention (IR) (Breitbart *et al.* 1987). The prevalence of AS events varies among different species. In humans, 95% of genes undergo alternative splicing (Pan *et al.* 2008; Wang *et al.* 2008) while only 25% of genes in *C. elegans* show alternatively splicing (Ramani *et al.* 2011). Sammeth and colleagues studied the extent of different types of AS events in 12 different metazoan genomes and showed that ES events are more prevalent in vertebrates than in invertebrates, while IR events are more abundant in invertebrates (Sammeth *et al.* 2008). A more comprehensive analysis on 65 species sampled major eukaryotic lineages including early branching animals, showed the significant increase in ES events in bilaterians compared to all other eukaryotic groups. (Grau-Bové *et al.* 2018). The analysis only examined the annotated exons for each species and this may introduce bias due to differences in annotation completeness in different species. Several studies have examined alternative splicing in different plant species. About 40% of intron-containing genes undergo alternative splicing in *Arabidopsis thaliana* (Filichkin *et al.* 2010) and *Camellia sinensis* (Zhu *et al.* 2018), while in maize and soybean, this number is close to 65–70% (Shen *et al.* 2014; Thatcher *et al.* 2014; Iñiguez *et al.* 2017). Among the different AS types, IR was found to be most abundant in the plant species. This is also supported by another study that quantified ES and IR frequencies across 65 eukaryotic lineages and observed reduced ES-to-IR ratio in plants and chlorophytes compared to vertebrates and bilaterians (Grau-Bové *et al.* 2018) Together, these studies show that AS is common among all eukaryotic species but that there are differences in the proportion of different classes of AS events.

In order to fully capture the evolutionary changes in AS patterns, it is important to obtain a comprehensive map of AS events of distantly related taxa. For this purpose, *Chlamydomonas reinhardtii* offers a unique position in the eukaryotic phylogeny. It is one of the early diverging lineage leading to plants that also shares important features with the Last Eukaryote Common Ancestor (LECA) (Rogozin *et al.* 2009; Cross and Umen 2015). This study aims to capture the extent of AS events in *C. reinhardtii* and the proportion of different AS classes. About 88% genes in *C. reinhardtii* are multi-exon genes with an average number of 6.3 introns per gene (Labadorf *et al.* 2010; Lin *et al.* 2018). Previous studies utilized EST data to identify the AS events in *Chlamydomonas* (Labadorf *et al.* 2010; Raj-Kumar *et al.* 2017). Analysis of seven million EST sequences found that about 20% of genes (3342 out of 17,746 genes) show alternative splicing in *Chlamydomonas*, and that IR is the most prevalent form, which contributes about 40% of the total AS events. A better estimate of AS events and their prevalence can be obtained using RNA sequencing methods. Thus, we utilized previously reported longitudinal RNA-seq data derived from cell cycle synchronized *Chlamydomonas* cells, to obtain a more complete repertoire of AS events in *C. reinhardti*i (Zones *et al.* 2015).

*Chlamydomonas* cells exhibit strong diurnal synchrony when grown in alternating light-dark conditions (Cross and Umen 2015). To capture the transcriptional pattern during a diurnal cell cycle, RNA from synchronized *Chlamydomonas* cells in a 12 hr light and 12 hr dark cycle were obtained from 28 time-points from one cycle with two independent biological replicates. The timepoints are named from 1 to 24 and the numbers correspond to the number of hours from the onset of illumination. During the light to dark transition, additional timepoints at 30 min intervals were taken between 11 to 15 hr. An average of ten million paired-end reads of length 101 bp were obtained for each sample (4.1 – 39.5 million reads). Using this transcriptomic data, distinct expression patterns of genes involved in different biological pathways and their temporal ordering during the diurnal cycle were identified. About 80% of the transcripts showed differential expression during the diurnal cycle (Zones *et al.* 2015). However, the study did not examine changes in splicing during the diurnal cycle. We used this transcriptome data to identify and characterize the AS events during the diurnal cycle. We developed a novel pipeline to quantify the IR events, and these events are analyzed separately from other AS events. Further, we identified the AS events that show periodic changes during the diurnal cell cycle and analyzed a subset of the AS events that introduce a premature termination codon (PTC) or affect an annotated domain.

## Materials and Methods

### RNA-seq data pre-processing and two-pass alignment

The RNA-seq data were obtained from NCBI Gene Expression Omnibus repository under accession number GSE71469 (Zones *et al.* 2015). The reads were trimmed and filtered using Trimmomatic (v33) (Bolger *et al.* 2014) with standard parameters to remove low quality reads and Illumina adapter sequences. Reads shorter than 60bp were removed.

The filtered reads were aligned to the *Chlamydomonas* genome (v5.5) using the STAR program with two-pass alignment (Dobin *et al.* 2013). In this approach, splice junctions (SJ) identified in the first alignment are used for annotation in the second alignment. Previous studies showed that this approach improved the identification and quantification of SJs by performing low stringency alignment that results in higher sensitivity (Veeneman *et al.* 2016). The STAR alignment program was used for both the first and second alignment run using standard parameter except the following changes 1) the minimum overhang length (–outSJfilterOverhangMin) was changed to 8 for SJ detection to enhance the stringency, 2) all read alignments were end to end type (–alignEndsType) to avoid soft trimming, and 3) the minimum intron length (–alignIntronMin) and maximum intron length (–alignIntronMax) were changed to 10 and 3000 respectively. This range covers more than 95% of all intron lengths in the version 5.5 of the *Chlamydomonas* genome. Other studies that analyzed RNA-seq data of *Chlamydomonas* also used the given intron length paramter (Zones *et al.* 2015). The alignment files for each sample in SAM format are sorted, indexed and converted to BAM format using Samtools (Li *et al.* 2009). The parameters of STAR alignment were set to identify the occurrence of new splice junctions. This can also result into identification of spurious splice junctions. Thus, to reduce the number of potential false positives, a set of filtering criteria were applied for SJ selection. The filtering criteria are: 1) The SJ must be present in both replicates of at least one timepoint under analysis, 2) The SJ must be supported by at least two reads, and 3) The SJ must not lie within a repeat region (the coordinates of repeat regions of *Chlamydomonas* genome v5.5 were obtained from Phytozome v12). The stepwise version of these command lines and a bash script to obtain these command lines for multiple samples are available at the Github repository.

### Quantification of splicing events using MAJIQ

The aligned BAM files are used to identify alternative splicing (AS) events using MAJIQ program (Vaquero-Garcia *et al.* 2016). MAJIQ identifies and quantifies local splicing variation (LSV) from the given set of aligned RNA-seq reads. It captures both the *de novo* and annotated SJs and quantifies them using the Percent Spliced In (PSI) metric where it assigns a PSI value to each junction in the given AS event. PSI value reflects the proportion of reads mapping to one junction compared to others in the AS event, and thus, provides a probability for each AS, ranging from 0 to 1. MAJIQ uses a builder command to build a splice graph that consist of all splice junctions. The BAM files were provided along with the information of replicates for timepoints and *Chlamydomonas* genome sequence (v5.5) to the MAJIQ pipeline, and it built the splice graph using this information. Each SJ in the splice graph is quantified for the sample by calculating the PSI value of the junction in the given splicing event. This step is performed using MAJIQ psi command line with default parameters. The MAJIQ output is then summarized in a tab delimited file for each sample using MAJIQ voila command line. The MAJIQ program ran successfully for all timepoints except for timepoint 13, where the program did not converge and did not give any error. Thus, we excluded this timepoint from further analysis. We also obtained read counts of each SJ using an SQL script to parse the splicegraph generated by MAJIQ. This script was provided by Christopher Green, author of the MAJIQ-SPEL (Green *et al.* 2018).

The *.tsv files and the readcount file of each sample are used to screen and annotate the junctions. Each SJ in a given splicing event was annotated as either “canonical” if it has highest average PSI value and the other splice junctions were annotated as “alternate”. Two criteria were applied to remove the noisy splicing events. The filtering criteria are 1) PSI value of at least one alternate SJ > 0.05, and 2) Number of reads mapping to at least one alternate SJ >= 2. We generated a matrix of PSI values with each alternate SJ as a row and each column as a time-point for the downstream analysis. Since more than 60% splicing events occur at just one timepoint, the PSI matrix is a sparse matrix with many null/missing data. Thus, a pseudo-count of 0.0001 replaced the null values. The implementation of the above mentioned criteria and matrix generation was performed in Python and the program is available at the Github repository.

### Quantification of intron retention (IR) events

To identify the IR events, a pipeline was developed that utilizes the same BAM files and quantifies them using Percent Intron Retention (PIR) metrics. This pipeline identifies IR events specifically in the constitutive introns that are not part of annotated exons based on genomic coordinates of *Chlamydomonas* genome v5.5 provided by Phytozome v12.

The IR pipeline takes the gff3 (General Feature Format) file, RNA-seq indexed BAM files and their replicate information as input. In this study, the pipeline utilized the *Chlamydomonas* genome gff3 file to obtain all exon coordinates along with the gene information, and then merged the exons that are overlapping. Using this information, the intron coordinates, 5′ and 3′ splice site were derived. The pipeline then filtered the mapped reads based on mapping quality score (MAPQ) (MAPQ >= 10). Using the coordinates, it identified the filtered reads in each bam file that are 1) reads that spliced out an intron (#SJ), 2) reads that mapped from the exon into the intron at 5′ splice site (#IE5), 3) reads that mapped from the exon into an intron at 3′ splice site (#IE3). A user-defined filter determined the number of mappable positions at the intron-exon junction that should be covered by a read in both the exon and intron (default: 10bp) The pipeline also calculated the coverage of each intron (Introncov) using Bedtools (Quinlan and Hall 2010). The read counts and the intron coverage were averaged across replicates. A set of filters was then applied to identify IR events in provided sample file. These filters were: 1) #SJ >= 5, 2) #IE3 >= 2 and #IE5 >= 2, 3) Introncov >= 1.0. PIR value was calculated for the filtered IR events with the following equation:

$$\text{PIR}=\frac{\#{\text{IE}}_{5}\;+\;\#{\text{IE}}_{3}}{\#{\text{IE}}_{5}\;+\;\#{\text{IE}}_{3}\;+\;\#\text{SJ}}$$

Introns with at least five spliced reads (#SJ >= 5) but no intron – exon junction mapping reads are assigned a PIR value as 0.0. Introns with fewer than five spliced reads are regarded as insufficient coverage junctions and their PIR values are assigned as 0.0. The IR pipeline provides output is in a matrix format with IR events as rows and timepoints/samples as columns. This matrix was utilized for downstream analysis. This pipeline is implemented in Python and is available at the Github repository.

### Diurnal cell cycle transcriptome analysis

Both the PSI and PIR matrices were analyzed separately using K-mean algorithm implemented as kmeans function in R. To estimate the number of clusters in each matrix, the “elbow” method was applied (Sugar *et al.* 1998). In this method, the within-cluster sum of squares, also called intra-cluster variation, was calculated and plotted as the value of k ranged from 1 to 20. This plot helps to obtain the appropriate number of clusters based on change in the intra-cluster variation as the number of cluster increases. In this study, the clusters were obtained for k-range of 3 to 10. The kmeans function in R assigned each row that corresponds to the AS event to the nearest cluster such that the total intra-cluster variation is minimized (Tarpey 2007). The kmeans output provided a summary of the cluster size and centroid value for each cluster at each timepoint that is the representative of a given cluster. The centroid values were then used to cluster the time-points using hierarchical clustering and was plotted using pheatmap package in R. In both PSI and PIR matrices, clustering was performed after removal of missing values in the dataset. PIR was not calculated for SJ at the timepoints that were missing sufficient reads, and thus these events were removed for the k-mean analysis.

The clusters obtained from kmeans clustering were then analyzed individually for the change in PSI/PIR pattern and change in gene expression during the diurnal cell cycle. The gene expression data of each gene in RPKM metric was obtained from the Supplemental Data 1 and 2 (Zones *et al.* 2015). The genes were binned based on the cluster assignment and both the expression data and PSI/PIR values were independently analyzed to obtain the mean, standard deviation, standard error and confidence interval of each time-point for each cluster. The mean along with standard error as error bars was plotted using ggplot2 in R to interpret the changes in the PSI/PIR values and gene expression of each cluster during the diurnal cell cycle. This analysis was implemented in R and is available at the Github repository.

Each alternate SJ was classified as a frame-preserving or a frame-disrupting event by calculating the difference in number of bases that differ from the canonical splice junction at the alternate site, and then identifying whether this difference is divisible by 3. This information was then combined with the average PSI value of the alternate SJ to test the hypothesis that frame-preserving events occur at higher frequency than the frame-disrupting events. We tested this hypothesis using Wilcoxon rank sum test (significant for p-value < 0.01) with Bonferroni correction, implemented in R statistical package. The code to classify the AS events as frame-preserving or frame-disrupting was implemented in Python and the hypothesis testing was performed in R.

### Gene-specific de novo transcript construction using Trinity

The genes in specific clusters that showed a coordinated splicing pattern with the diurnal cell cycle were further analyzed for their known Pfam domains and their potential function during the diurnal cell cycle. The effect of AS on the transcript of the gene was assessed by constructing the *de novo* transcripts. Both the canonical and alternative transcripts are compared and analyzed for their coding potential, change in the amino acid sequence and whether the AS event affect known domains. To this end, the maximum and minimum PSI/PIR value timepoints for the gene of interest were identified. Using the RNA-seq indexed bam files of these timepoints, the reads that map into the gene and their paired-end mates were obtained and saved in a fastq format. The fastq file was processed using Trinity (Haas *et al.* 2013) with default parameters with the minimum contig length set to 150 bp. The code to obtain the paired-end reads mapping for a specific gene was implemented in Python and will be available at the Github repository. All contigs that were at least 500 bp or longer were then translated into the protein sequence using ExPASY translate tool (https://web.expasy.org/translate/). The protein sequences obtained from canonical and alternative transcripts were pairwise aligned to analyze the changes in the protein sequence. The translated protein sequences were also aligned against non-redundant protein sequence database using BLAST (Altschul *et al.* 1997) to identify potential hits and conserved regions.

An indexed insertional library in *Chlamydomonas* is available with the insertion sites identified by sequencing (Li *et al.* 2016). Disrupted genes can be identified and we utilized this information to infer if alternatively spliced genes are potentially important for *Chlamydomonas*.

The implementation of all codes mentioned in the Methods sections is available at https://github.com/mpandeyWU/diurnalCycleCodes.

### Data availability

No data were generated for this manscript. Supplemental material available at figshare: https://doi.org/10.25387/g3.12759830.

## Results and Discussion

### Quantification of splicing changes in C. reinhardtii

**Figure 1:**
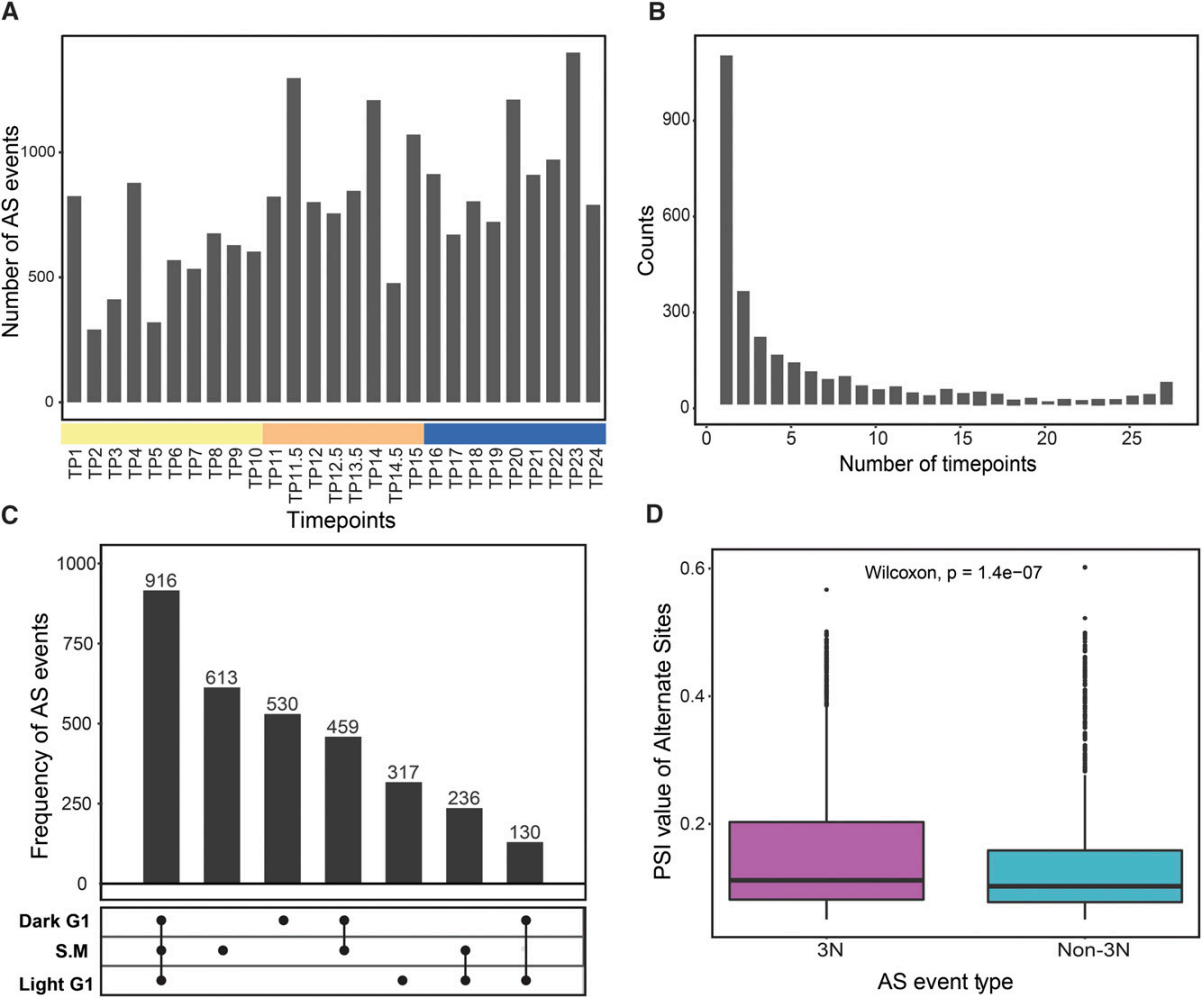
Distribution of alternative splicing events. (A) Frequency of alternative splicing events across all timepoints. Timepoints on the x-axis are color-coded by three phases: Light G1 timepoints (yellow), S-M phase timepoints (orange) and Dark G1 timepoints (blue). (B) Histogram showing the number of timepoints that where each SJ was observed. The left-skewed distribution suggests that most of the AS events are unique to a specific timepoint (C) Upset plot depicting the overlap between AS events in Light G1, S-M and Dark G1 phase based on the alternate splicing events. (D) PSI value distribution of frame-preserving (3N) and frame-disrupting events (non-3N) (p-value: 1.4 × 10^−7^ (Wilcoxon rank-sum test)).

The raw RNA-seq reads from 56 samples were mapped to the *C. reinhardtii* genome (Phytozome v12) (See Methods) and the resulting alignment files (*.bam files) were used to build a splicegraph with the MAJIQ program (Vaquero-Garcia *et al.* 2016), to quantify the splice junctions (SJ) at each timepoint. A total of 177,811 unique SJ were detected across all timepoints. The MAJIQ program identifies 37,925 AS events from the *Chlamydomonas* diurnal cycle transcriptome data. The Percent Spliced In (PSI) value that ranges from 0 to 1, was calculated; this value reflects the proportion of reads associated with each SJ in the given splicing event. The SJ in each alternative splicing event was classified as canonical or alternate based on the mean PSI value across all timepoints. The canonical site will have the highest mean PSI value and the remaining SJ are alternate sites. Requiring a PSI > 0.05 at one or more timepoints reduces the number to 13,288 AS events. The read counts for each SJ were also averaged across replicates at each timepoint. Only splicing events with more than 2 reads mapped at their alternate site at any timepoint were retained. This filtering results in 3220 AS events in 2281 genes. The presence and absence of these AS events at different timepoints showed no significant enrichment at any specific timepoint (Figure 1A). The histogram of distribution of alternate splice site occurrence across different timepoints suggest that most of these splicing events are unique for any given timepoint and only a small fraction of these sites occur at all timepoints (Figure 1B).

G1 occurs during both the light and dark phase in light synchronized *Chlamydomonas* cultures. The S-M phase occurs during the light to dark transition and early in the dark phase (Cross and Umen 2015). For analysis purposes, the timepoints are divided into three categories. The TP1 to TP10 timepoints define the Light G1 phase, TP11 to TP15 define the S-M phase and TP16 to TP24 define the Dark G1 phase. Twenty-nine percent of the splicing events occur in all three phases (n = 916). Twenty percent of unique AS events occur in the S-M phase (n = 611) and 17% the dark G1 phase (n = 549) (Figure 1C). These two phases share 461 AS events that do not occur in the Light G1 phase. The Light G1 phase shows the smallest number of unique AS events (n = 315) and a lower number of events shared with S-M phase and Dark G1 phase (n = 229 and n = 132, respectively).phase (n = 229 and n = 132, respectively).

The *C. reinhardtii* genome release (v5.5) in Phytozome v12 reports 1785 alternatively spliced transcripts in 1529 genes. Comparison between AS genes identified in this study and Phytozome reported AS genes showed only 620 genes in common. There are 909 genes that are unique to Phytozome and 1661 genes unique to this study. This low overlap could be due to difference in the culture conditions in which *Chlamydomonas* cells are grown. The novel AS genes in the Phytozome data are likely due to the fact that many of them are derived from different culture conditions such as nitrogen starvation, copper deficiency, bilin signaling and others, whereas the diurnal cycle data were from cells grown in constant media.

### Frame-disrupting events have low relative abundance compared to the frame-preserving events

The effect of the alternative splicing events on the predicted proteins was analyzed for the 3220 events. The splicing events with more than one significant alternative SS (n = 253) were classified as complex events and not evaluated further. The remaining 2967 alternative splicing events were classified as frame-preserving or frame-disrupting events by calculating the difference in base pairs of the canonical and alternate splice site, and if this difference is divisible by 3. If the alternative SS skips a known exon, it was classified as a putative ES event, and exon length is tested if it is divisible by 3 or not. Interestingly, both types of alternative splicing events are nearly equally prevalent in the *Chlamydomonas* transcriptome. There were 1412 frame-preserving and 1555 frame-disrupting events. We compared the PSI value associated with these events and found that the frame-disrupting events show a significantly lower PSI value compared to the frame-preserving events (p-value = 1.4 × 10^−7^, test: Wilcoxon test (Bonferroni’s correction)) (Figure 1D). This suggests that frame-disrupting events either occur at a low frequency or those transcripts are subjected to nonsense mediated decay (NMD) (Celik *et al.* 2017; Lin *et al.* 2018). We further investigated the frame-preserving events if they introduce the stop codon in the alternate transcript. Out of 1412 frame-preserving AS events, 624 events add sequence region in the primary transcript by either alternate 5′ or alternate 3′ splicing (Supplemental Data 3). We translated this added sequence region with all six potential ORF, and found 105 events that introduce stop codon in all six frames, most of them in the longest inserted sequences (Supplemental Figure S8). This suggests that a small proportion of frame-preserving events (nearly 7%) may also be subjected to NMD.

### PSI clustering reveals transcripts with a coordinated splicing pattern in the diurnal cycle

**Figure 2:**
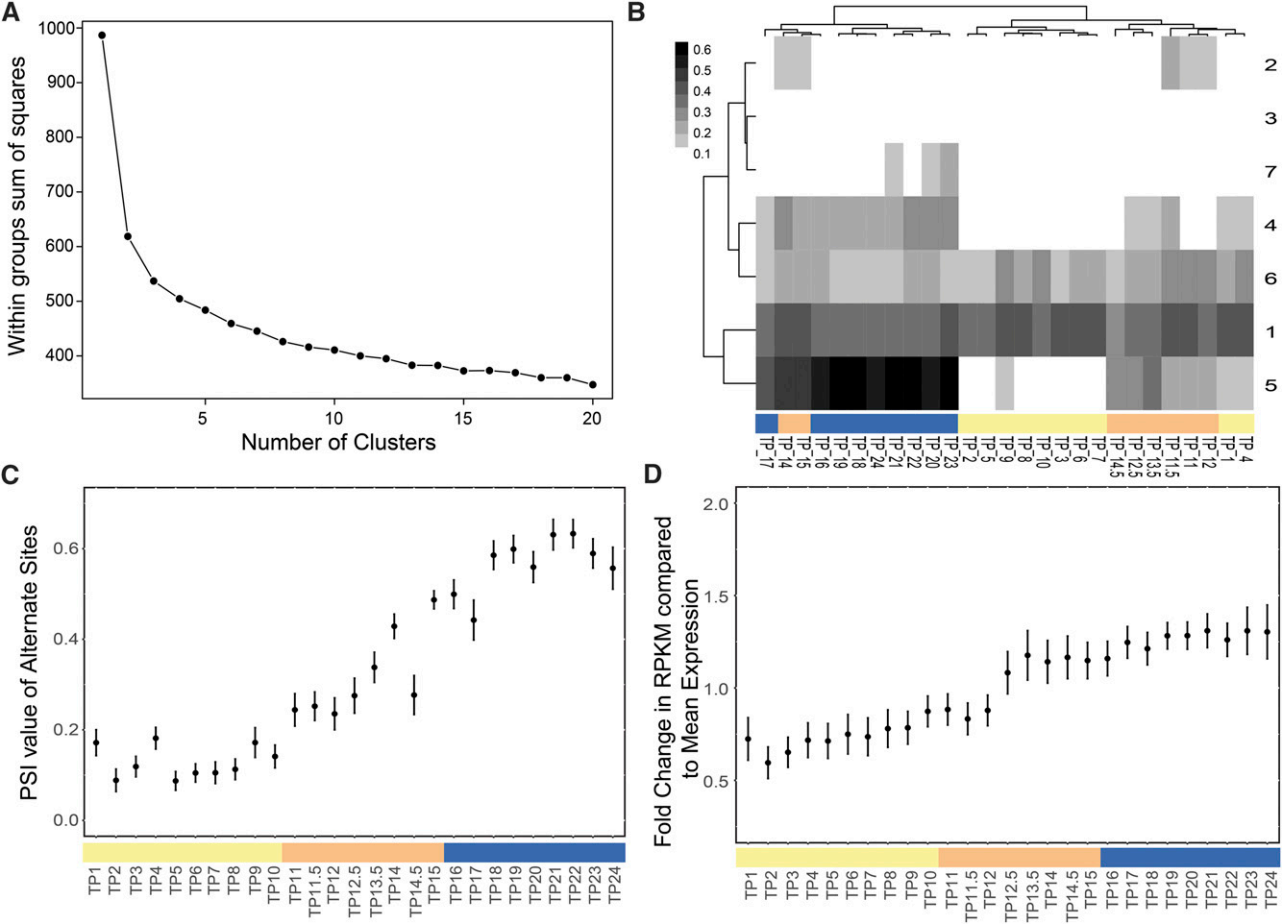
Cluster analysis along the diurnal cycle. (A) Sum of squares calculated within each cluster as a measure of variation is (Intra-cluster variation) plotted to estimate the number of clusters for k-mean clustering (B) Heatmap of the PSI values of filtered alternative SS The white to black transition on the heatmap represents PSI value that ranges from 0 to 0.6. The numbers on the right of the heatmap are cluster numbers referred to in the text and x-axis are timepoints color-coded by three phases. (C) Mean PSI value of Cluster 5 alternative SS events are plotted for each timepoint (bar represents color code by phase: light G1: yellow, S-M: orange, dark G1: blue) where dot represents the mean PSI value across genes and error bars represent standard error. (D) Fold change in the RPKM value of Cluster 5 genes. compared to their mean expression value across all timepoints (bar represents color code by phase: light G1: yellow, S-M: orange, dark G1: blue). The dot represents the mean fold change across genes and error bar represents standard error.

More than 80% of the *Chlamydomonas* transcriptome, at one or more timepoints, show at least a twofold change in expression from the mean expression value and those genes were clustered based on their expression pattern (Zones *et al.* 2015). To identify AS events with substantial variation during the cell cycle, the filtered alternative splicing events (n = 2967) were clustered based on the PSI value of the alternative SS, using k-means algorithm, implemented as kmeans function in R (Supplementary Data Table 1) (See Methods). Since most alternate SS are unique to specific timepoints, we observe a sparse PSI matrix of alternate SS by timepoints with many null values. Thus, a pseudo-count of 0.0001 was added to all entries to account for these null values. The number of clusters (k) was estimated by plotting the total variation within each cluster (intra-cluster variation) as a function of a range of k values (1 to 20) (Figure 2A) (See Methods). Although there is no sudden decrease in the total variation, the curve linearizes in the k-range of 5 – 10. We performed k-mean clustering on the data with k-value ranging from 3 to 10 (Supplementary Figure S1A and B). The kmeans function returns the centroid PSI value for each timepoint that is representative of the given cluster. Using these centroid values of clusters, the timepoints were clustered using hierarchical clustering and a heatmap of PSI values (Figure 2B) was generated using pheatmap package in R (See Methods). Among these clusters obtained from k-values 3 to 10, we found that the k-means clustering output obtained by k = 7 appeared most biologically relevant because there is strong segregation of Dark G1, Light G1 and S-M phase timepoints with 30 to 1917 alternate SS in the clusters. Cluster 1 with 60 alternate SS, cluster 6 with 135 alternate SS and cluster 3 with 1917 alternate SS represent sites that have consistently high, medium and low PSI values, respectively, throughout the diurnal cycle (Figure 2B). Cluster 2 (n = 313) also shows a consistently low PSI value except for a few timepoints. Cluster 7 (n = 348) and cluster 4 (n = 164) show a relatively high PSI value during the Dark G1 timepoints compared to the S-M distinct pattern with low PSI values at Light G1, medium PSI values at S-M phase and high PSI value at Dark G1 stage.

**Table 1:** Alternatively spliced Cluster 5 genes

Gene name	AS event type	Pfam ID	Pfam ID description	Effect of alternative splicing on annotated domain
Cre01.g051700	ES	PF13639	Ring finger domain	Not affected
Cre02.g081176	Alt 3′	PF00588	SpoU rRNA methylase family	Introduce 1aa in the rRNA methylase domain
Cre03.g146487 (XPO1)	ES	PF03810	Importin-beta N-terminal domain	Introduces a PTC that disrupts the Importin N-terminal domain
		PF08389	Exportin 1-like protein	
		PF08767	CRM1 C terminal	
Cre03.g159500 (ODC1)	ES	PF00278	Pyridoxal-dependent decarboxylase, C-terminal sheet	Introduces a PTC
		PF02784	Pyridoxal-dependent decarboxylase, pyridoxal binding	
Cre05.g242850	Alt 3′	—	—	—
Cre06.g278239 (ASF/SF2)	Alt 3′	PF00076	RNA recognition motif (RRM)	Introduces a PTC after RRM domain
Cre06.g282000 (STA3, SSS3)	Alt 3′	PF00534	Glycosyl transferases group 1	Not affected
		PF08323	Starch synthase catalytic domain	
		PF16760	Starch/carbohydrate-binding module	
Cre07.g355050	Alt 5′	—	—	—
Cre08.g375084 (RBM25)	Alt 3′	PF00076	RNA recognition motif (RRM)	Introduces a PTC
		PF01480	PWI domain	
Cre09.g392000	ES	PF09763	Exocyst complex component Sec3	Not affected
Cre09.g393900	ES	PF00925	GTP cyclohydrolase II	Not affected
Cre09.g400330	Alt 5′	PF00069	Protein kinase domain	Not affected
Cre09.g413114	Alt 5′	—	—	—
Cre10.g418500	ES	—	—	—
Cre10.g441350	Alt 3′	—	—	—
Cre11.g480700	Alt 3′	—	—	—
Cre13.g586916 (SRp20)	Alt 5′	PF00076	RNA recognition motif	Disrupts RRM domain; Introduces a PTC
Cre14.g615224	Alt 3′	—	—	—
Cre16.g659667	Alt 5′	—	—	—
Cre17.g710800	ES	PF01106	NifU-like domain	Not affected
Cre17.g722100	Alt 3′	PF04727	ELMO/CED-12 family	Not affected
Cre17.g726700	Alt 3′	PF00501	AMP-binding enzyme	Not affected
		PF13193	AMP-binding enzyme C-terminal domain	
		PF16177	Acetyl-coenzyme A synthetase N-terminus	
Cre17.g733150 (COP11,COP12)	ES	PF00072	Response regulator receiver domain,	Introduce 59 aa in the chlamyopsin domain
		PF00512	His Kinase A (phospho-acceptor) domain	
		PF01036	Bacteriorhodopsin-like protein (chlamyopsin)	
		PF02518	Histidine kinase-, DNA gyrase B-, and HSP90-like ATPase	
Cre17.g745847	ES	—	—	—
Cre17.g746597 (SerS10)	ES	PF00450	Serine carboxypeptidase S10	Introduce 19aa in the peptidase S10 domain

These splicing events follow a coordinated splicing pattern with the diurnal cell cycle (Figure 2C), and thus, we followed up on these events. Since changes in the gene expression could affect the detection of a particular SS at a given timepoint, we asked if this coordinated splicing pattern is related to differential gene expression across these genes. The RPKM values of these 26 genes (Zones *Et Al*., 2015; Supplementary Dataset 1 and 2) across all timepoints were normalized by their mean expression value to give a fold-change in gene expression compared to the mean expression value. The average value of the fold-change of these genes and their standard deviation is plotted for all timepoints (Figure 2D). Although there is trend of increased gene expression among these genes at the Dark G1 timepoint compared to the Light G1, the mean fold change across these genes from their mean expression value is less than twofold at all timepoints (Figure 2D). This suggests that most of these genes do not show large and changes in gene expression and that AS is regulated independently of gene expression.

### PSI Cluster 5 events and their effect on the transcript

Of the 30 splicing events in Cluster 5, there are 5 alternative 5′ and 11 alternative 3′ splicing events. Six splicing events occurs as complex events that are paired and were collapsed into three different ES events. Seven other splicing events were found to skip one or more annotated exons and were also classified as ES events. Thus, in total, ten ES events were detected in this cluster. One splicing event could not be classified into any of three categories and was excluded for analysis purposes. In the final filtered dataset, there are 26 alternative splicing events in 25 genes, where one gene (Cre10.g418500) has two independent alternative splicing events. These events are depicted in Supplementary Figures S2 to S5 and described in Table 1.

**Figure 3:**
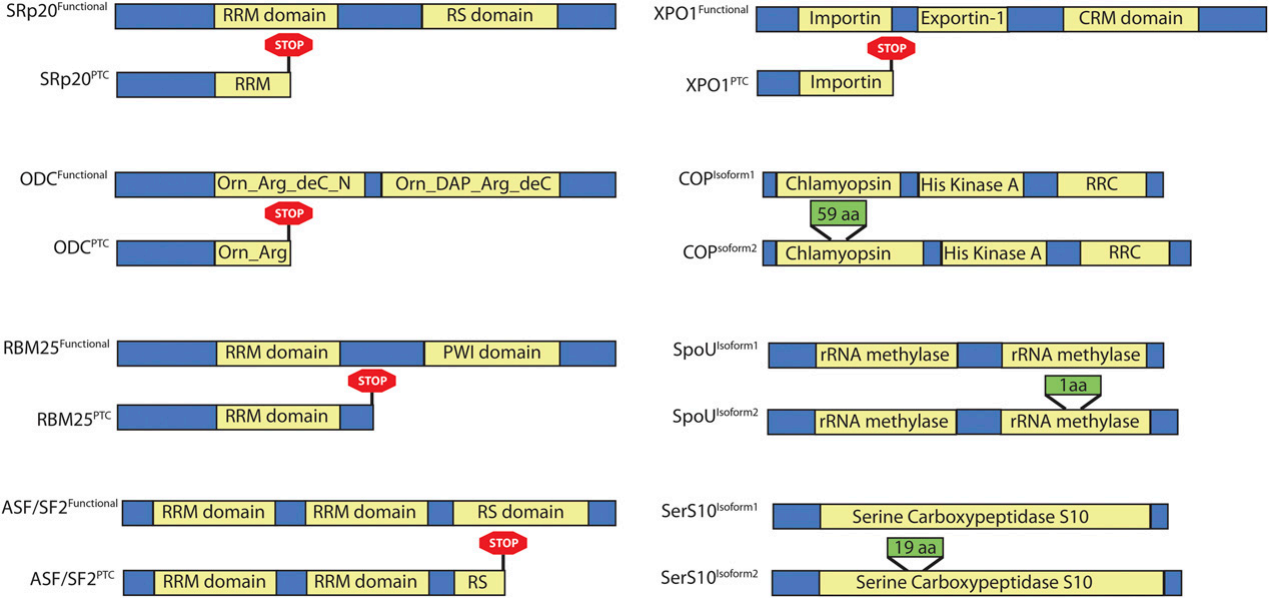
Effect of Cluster 5 AS events on the protein sequence, specifically those events that either introduce PTC in the transcript or affects the annotated domain. The yellow boxes depict identified Pfam domain region in the protein sequence, and rest of the protein sequence is depicted in blue. Green boxes show insertion in the protein sequence due to the AS event, and consists the number of amino acids inserted. The red box shows the insertion of stop codon in the sequence due to AS event.

The twenty-five genes that make up PSI Cluster 5 and show coordinated splicing pattern with the diurnal cell cycle are not enriched for any specific Gene Ontology (GO) terminology. Sixteen alternatively spliced genes have a known domain from Protein Families (Pfam) database (El-Gebali *et al.* 2019). To evaluate the effect of the splicing events on the protein sequence of these 16 genes, the two timepoints that show highest and lowest PSI value for the alternate site were picked. We collected the paired-end reads mapped at these genes in the two samples and obtained their gene-specific *de novo* transcripts using Trinity (Haas *et al.* 2013) for both samples (See Methods). The transcripts that were at least 500 bp long were translated into amino acid (aa) sequence from six potential ORFs. In both samples, the amino acid sequences are manually analyzed for their identity to known Phytozome protein sequence of the gene and whether they are product of the AS event under analysis. The canonical and alternative transcript along with their aa sequence aligned using global sequence alignment tool NEEDLE (https://www.ebi.ac.uk/Tools/psa/emboss_needle/). Eight of the 16 splicing events either introduce a PTC in the transcript or affects the annotated domain (Figure 3). The alternative transcript produced by five of these events is annotated in Phytozome v12. Some of these genes are well studied and their splicing pattern shows high concordance with their activity or with the homologs in other species. Introduction of PTC in the transcripts and changes in the annotated domain suggests that a subset of PSI Cluster 5 genes are actively regulated during the diurnal cell cycle in *Chlamydomonas*. The eight examples shown in Figure 3 are described in the following sections.

### Coordinated changes in splicing pattern of ornithine decarboxylase 1 (ODC1) during light-dark transition

Polyamines are ubiquitously present polycations that play important roles in cell growth and division in both prokaryotes and eukaryotes (Minois *et al.* 2011). They are important for sequestration of the negative charges of DNA and RNA, actin filament formation during spindle formation and cytokinesis (Tassoni *et al.* 2018). Polyamine levels in cells are regulated by their biosynthetic enzymes (Gemperlová *et al.* 2006). Putrescine is an important polyamine that is biosynthesized directly from ornithine by ODC (E.C: 4.1.1.17) or indirectly from arginine by arginine decarboxylase (ADC) (E.C: 4.4.4.19). The AS of *ODC1* gene (Cre03.g159500) show coordinated splicing pattern with the diurnal cycle.

**Figure 4:**
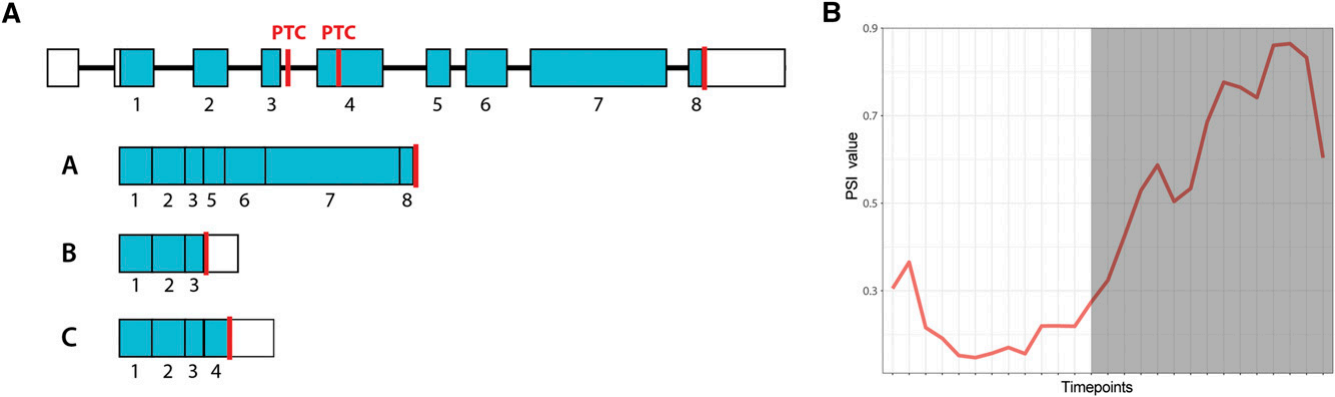
Alternative splicing of *ODC1* gene. (A) Genetic structure and three isoforms of the *ODC1* gene. Isoform A encodes a functional transcript. Isoform B and isoform C introduce in-frame stop codon (white box: UTR, blue box: CDS, black line: introns, red line: stop codon). (B) Change in the PSI value of isoform C during the diurnal cell cycle where white refers to timepoints taken in light, and gray refers to timepoints taken in dark.

AS in *ODC1* gene produces three transcripts. Isoform A skips exon 4, which contains an in-frame stop codon in the transcript, to produce a transcript that likely encodes for functional ODC1 enzyme. The other two isoforms introduce an in-frame stop by retention of intron 4 (Isoform B) or inclusion of exon 4 (Isoform C) (Figure 4A). The AS event that includes or excludes the exon 4 show differential splicing pattern during the cell cycle. The non-functional isoform C shows a low PSI value (TP6 = 0.147) during the light phase, but as cells enter into the dark phase, the PSI value of Isoform C increases to 0.864 at TP21. On the other hand, the functional isoform A shows a high PSI value during the light phase (TP6 = 0.853) and declines to low levels in the dark phase (TP21 = 0.136) (Figure 4B). Previous studies showed the light-mediated activation of the ODC1 enzyme in *Chlamydomonas* cells and tobacco plants (Voigt *et al.* 2000; Gemperlová *et al.* 2006). Voigt and colleagues showed a rapid increase in ODC activity from 10 to 75% of maximum within an hour when *Chlamydomonas* cells are transferred from dark to light conditions. This observation is concordant with the changes in the alternative splicing pattern as PTC-containing transcripts are enriched in the dark phase. The authors also found that transcription inhibition does not completely block the ODC activity but reduces it to 30% in the presence of light, however translation inhibition completely blocks the ODC activity. Another study by the same group also showed that there is no significant change in the RNA level when *Chlamydomonas* cells are treated with spermidine, however ODC activity declines (Theiss *et al.* 2002; Voigt *et al.* 2004). These observations, along with the differential splicing pattern of *ODC1* gene found in this study, suggests a multi-level regulation, both at post-transcriptional and translational level, to fine tune the expression of *ODC1* gene.

### AS pattern of SR protein coding transcripts during diurnal cell cycle

SR proteins play a crucial role in recruitment of the spliceosome components to the splice site and act as splicing enhancers (Shepard and Hertel 2009). In addition, they are also involved in nuclear transport of mRNA, NMD and mRNA translation. SR proteins are identified by their RNA recognition motif (RRM) domain followed by a Serine-Arginine rich region called an RS domain (Shepard and Hertel 2009). Most SR protein families (SRp20, SRp38, p54, 9G8, SRp75, SRp55, SRp30c, SRp40, ASF/SF2 and SC35) have unproductive splicing isoforms that introduce a PTC in the SR transcript in humans and mice (Lareau *et al.* 2007). Several SR proteins use unproductive splicing to maintain their homeostatic expression level (Jumaa and Nielsen 1997). Lareau *Et Al*., (2015) showed that the unproductive splicing in some SR proteins is evolutionarily conserved. For example, SRp40 orthologs in *Drosophia melanogaster*, sea urchin, *Neurospora crassa* and *Aspergillus niger* produce PTC containing transcripts (Lareau and Brenner 2015).

In Cluster 5, two SR proteins are alternatively spliced and the AS events introduce an in-frame PTC in the sequence (Figure 3). They are the SRp20 family gene (Cre13.g586916) and the ASF/SF2 family gene (Cre06.g278239). The inclusion of an intron in the SRp20 family gene disrupts the RRM domain, while in the ASF/SF2 family gene, it disrupts the RS domain. The canonical isoform of the SRp20 family gene is predominantly expressed during the S-M phase, with a PSI of 0.95 at TP12.5. The PTC-introducing transcript has its highest PSI value during Dark G1 timepoint (TP23:0.86). The overall expression level of this gene varies in a similar way during diurnal cell cycle with average RPKM value of 132.6 during S-M phase and 57.75 during Dark G1 phase. This suggests that the AS transcript may undergo NMD. Interestingly, SRp20 homologs in mammalian cells autoregulate their transcript levels by inclusion or exclusion of an exon that introduces an in-frame PTC (Jumaa and Nielsen 1997). It is plausible that similar SRp20 autoregulation may be conserved in *Chlamydomonas*.

In the ASF/SF2 family gene, an in-frame PTC is introduced by inclusion of the last intron and affects the RS domain (Figure 3). Similar to the SRp20 family gene, the PTC-introducing transcript peaks during Dark G1 timepoints with a PSI value of 0.89 at TP22, while the canonical transcript peaks at TP12.5 with a PSI value 0.932. Sun and colleagues showed that the differential splicing pattern of the ASF/SF2 homolog in humans affects its cellular localization (Sun *et al.* 2010). The alternate transcripts of ASF/SF2 are retained in the nucleus, while the cononical transcripts are transported to the cytoplasm. This suggests that these alternate transcripts that are retained in the nucleus will neither be subjected to degradation by NMD, nor will be utilized for translation. The splicing pattern of ASF/SF2 gene in *Chlamydomonas* cells is similar to the human isoform that is retained in nucleus. And unlike SRp20, no significant change in the RPKM level of ASF/SF2 family gene in *Chlamydomonas* cells is observed.

*RBM25* (Cre08.g375084) and *XPO1* (Cre03.g146487) genes undergo AS during the diurnal cell cycle that introduces PTC in their transcripts (Figure 3). RBM25 is likely an RNA binding protein with an RRM domain and a PWI domain. XPO1 is likely a nuclear transport protein with an Exportin 1 like domain and a chromosome maintenance (CRM) domain. In the *RBM25* transcript, there is a sudden change in the PSI value of the PTC-including splice site from 0.272 at TP12 to 0.652 at TP12.5. In the *XPO1* transcript, the PTC-introducing splice site increases gradually during the post-mitotic phase with a peak at TP23 with a PSI value of 0.8226. In *Arabidopsis*, RBM25 controls the splicing of several transcripts and is required for the response to the phytohormone abscisic acid (ABA). Two independent *rbm25* mutants in *Arabidopsis* are hypersensitive to ABA treatment (Zhan *et al.* 2015). It will be interesting to find if RBM25 regulates the level of ABA response genes in *Chlamydomonas* as well (Yoshida 2005).

### AS events in transcripts that affect functional domains

**Figure 5:**
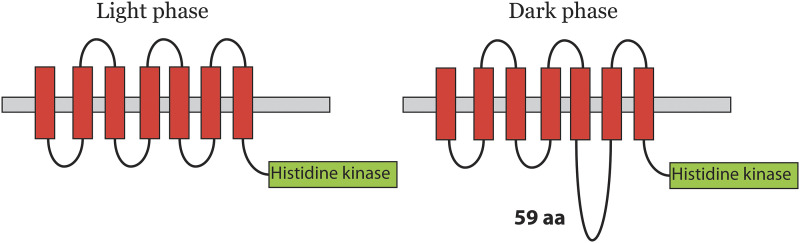
Alternative splicing of Cre17.g733150 introduces 59aa in the loop between helix 5 and 6 of Chlamyopsin domain during dark time-points in diurnal cell cycle.

Three AS events in Cluster 5 introduce changes in the transcript that alters annotated domains (Figure 3 and Table 1). Cre17.g733150 (COP11) encodes for a response regulator system that has seven helical *trans*-membrane (7TM) chlamyopsin domain (Bacteriorhodopsin-like protein) and a histidine kinase domain AS of this gene alters the 7TM domain. During diurnal cell cycle, an exon of length 177 bp is included in the transcript during the dark phase that affects the 7TM structure of the chlamyopsin domain (Figure 5).

Sequence comparison with the PFAM domain PF01036 shows that the protein sequence with the insertion has 59 aa between helix 5 and helix 6 whereas the protein sequence without insertion has all helices closely knitted together to make 7TM domain. It is likely that this insertion adds an intracellular loop between the two helices (Figure 5).

### Quantification of IR events in C. reinhardtii

IR events are the most prevalent form of AS in plants, unicellular eukaryotes and many invertebrates (Sammeth *et al.* 2008; Filichkin *et al.* 2010; Marquez *et al.* 2012). These events occur less frequently in vertebrates (Sammeth *et al.* 2008; Grau-Bové *et al.* 2018). This difference is consistent with the fact that vertebrates have much longer introns. (Sammeth *et al.* 2008; Gelfman *et al.* 2012). Previous studies on genome-wide identification of AS events in *C. reinhardtii* showed that IR is the most prevalent of AS, with 40% of the total AS events. In *Chlamydomonas*, MAJIQ identified only 268 IR events that were further reduced to 161 events after applying filters (PSI value of the alternate site > 0.05 and Read count at alternate site >= 2) (See Methods). This number is significantly lower than observed in previous AS analysis in *C. reinhardtii* (Labadorf *et al.* 2010; Raj-Kumar *et al.* 2017). A close analysis of these 161 IR events, identified by MAJIQ, showed that these events occur within annotated exon regions. Marquez and colleagues have differentiated such events from the IR events and referred them as exonic introns or exitrons (Marquez *et al.* 2015). “Constitutive introns” as referred by Marquez *Et Al*., 2015, are sequence regions that are defined as introns and are spliced out from pre-mRNA sequence to obtain the primary transcript. Retention of these introns in the alternate transcript is considered as IR event. Exitrons, on the other hand, are sequence region that are part of coding sequence in the primary transcript, but are introns (or spliced out) in the alternate transcript. It is unclear why MAJIQ does not capture IR in constitutive introns that are not part of any annotated exon. To capture the extent of IR in constitutive introns that are not part of annotated exons in *C. reinhardtii*, we developed a pipeline to identify and quantify the IR events using Percent Intron Retention (PIR) metrics (Braunschweig *et al.* 2014) (see Methods).

**Figure 6:**
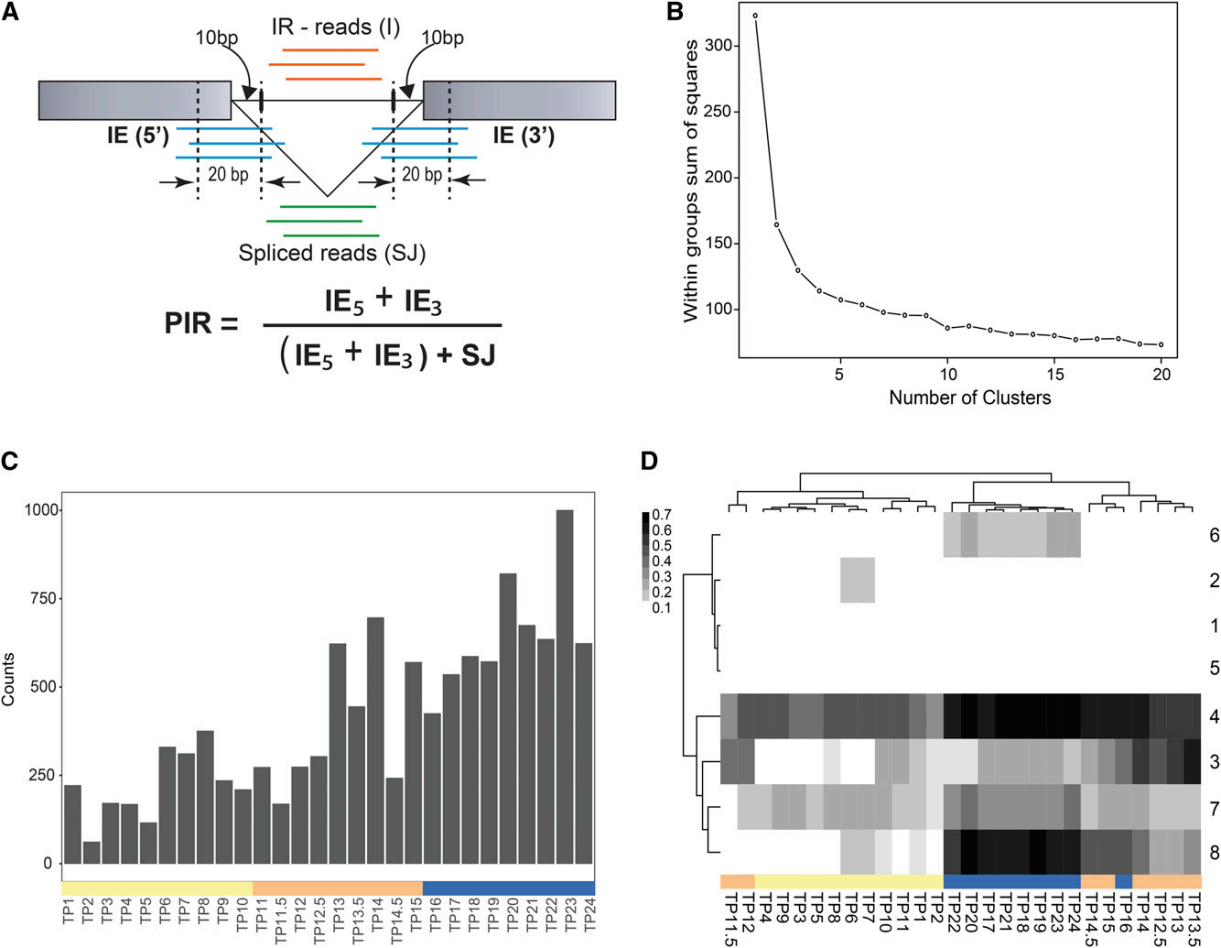
Cluster analysis of intron retention events. (A) IR events were quantified by identifying reads mapping at Intron-Exon (IE) and Exon-Exon (SJ) junctions and PIR is calculated by applying a range filters and using these read counts (See Methods for details) (B) Intra-cluster variation calculated for k range of 1 to 20 for IR events and plotted to estimate the number of clusters for k-mean clustering. (C) Frequency of IR events at different timepoints. Timepoints at x-axis are color-coded by phase: light G1 (yellow), S-M (orange) and dark G1 (blue). (D) Heatmap of the PIR values of filtered IR events. The white to black transition on the heatmap represents PIR value that ranges from 0 to 0.7. The numbers on the right of the heatmap are cluster numbers referred in the text.

The IR pipeline identifies the number of spliced reads for an annotated splice junction (#SJ), number of reads that map to the intron-exon junction at 5′ and 3′ end (#IE_5_ and #IE_3_) and calculates the coverage of reads mapping into the intron region (Intron_cov_) and number of reads mapping into the intron (#I) (Figure 6A). The read counts were averaged across replicates. Using this information, the IR pipeline filters out events and calculates PIR value for each IR event (see Methods). The pipeline identified 2149 IR events in 1679 genes based on the following criteria: 1) #SJ >= 5; 2) #IE_5_ >= 2 and #IE_3_ >= 2; 3) Intron_cov_>= 1.0 and #I >= 5; 4) PIR >= 0.05 for at least one timepoint in the cell cycle (See Methods). The pipeline assigned ‘NA’ to the introns that do not have sufficient reads for quantification (#SJ < 5) and adds a pseudo-count of 0.001 to rest of the PIR values (Supplementary Data Table 2). Unlike the other AS events quantified by PSI, the filtered PIR value showed an increased frequency of IR events during the Dark G1 timepoints (Figure 6C).

**Figure 7:**
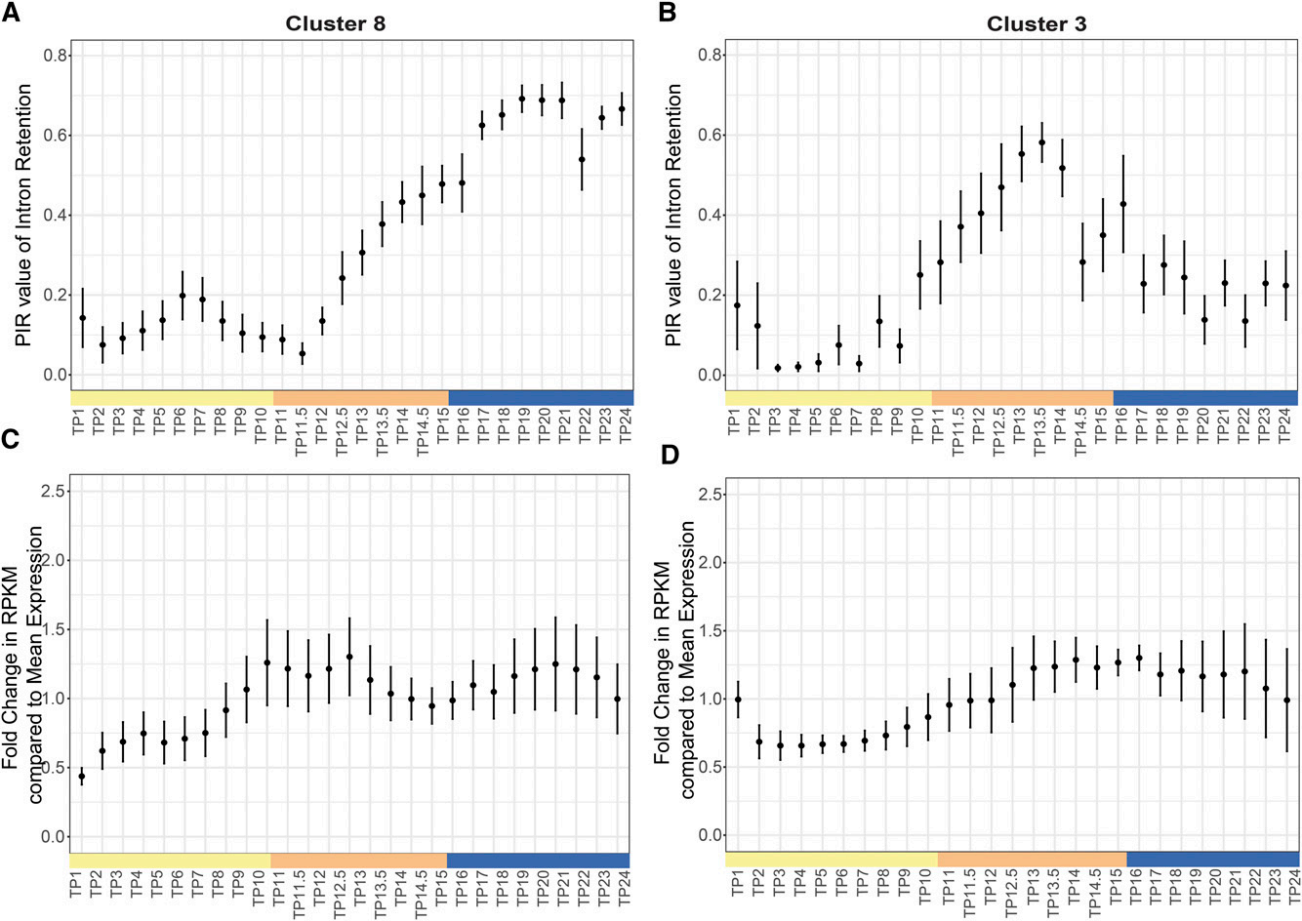
Time course analysis of Clusters 3 and 8. (A and B) Plot A and B depict mean PIR value of Cluster 8 and Cluster 3 IR events respectively, are plotted for each timepoint. The dot represents the mean value and the error bars show standard error at each timepoint (C and D) Plot C and D show mean fold change in the RPKM value of Cluster 8 and Cluster 3 genes respectively, compared to their mean expression value across all timepoints. Timepoints on x-axis are color-coded by phase Light G1 phase: yellow, S-M phase: orange, Dark G1 phase: blue.

**Figure 8:**
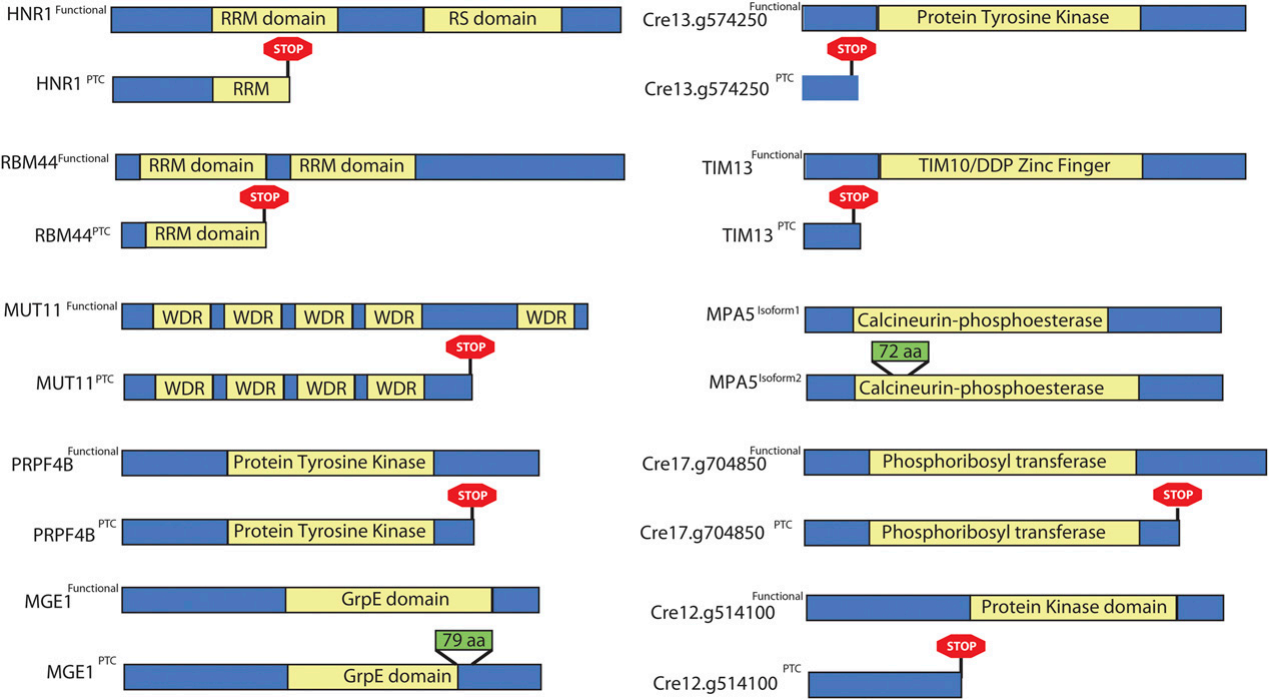
IR events that either introduce PTC in the transcript or affects the annotated domain.

By filtering out IR events with too few reads for quantification (See Methods), we reduced the number to 1025 IR events that were analyzed using k-mean clustering, implemented as kmeans in R. Similar to the PSI analysis, the number of clusters were obtained by plotting the intra-cluster variation as a function of k (1 to 20), where the curve linearizes in the k-range of 5 – 10 (Figure 6B). Thus, the clusters were obtained for the k values 3 – 10. The centroid PIR values for each timepoint were then used to cluster the timepoints using hierarchical clustering and a heatmap of PIR values was clustering output from k = 8 appears most biologically relevant (Figure 6D). The clusters obtained from k = 8 shows strong differentiation of light (TP1 – TP12) and dark (TP12.5 – TP24) timepoints. In the light timepoints, TP11.5 and TP12 are separated from TP1 - TP10, and in the dark timepoints, TP12.5 – TP16 are clustered separately from TP17 – TP24. This suggests that in addition to the primary separation of light-dark time points, there are secondary separations of the G1 and S-M time points. The number of IR events within each cluster ranges from 6 to 662. Cluster 1 and 5 represent events that have low PIR values throughout the cell cycle and constitute 80% of the IR events (n = 845). Cluster 4 with 14 IR events and Cluster 7 with 32 IR events show high and moderate retention rate throughout the cell cycle. Cluster 6 with 73 IR events shows a coordinated IR pattern with the light – dark cycle with introns that are retained during the dark timepoints. In contrast, for Cluster 2 the 45 IR events reflect IR during the light timepoints. However, the overall PIR value of IR events in these two clusters is low throughout. Clusters 3 and 8 show the strongest correlation with the cell cycle. Cluster 8 with 10 IR events in eight genes show an interesting pattern with low PIR value during Light G1 timepoints, moderate PIR values during S-M timepoints and high PIR values during Dark G1 timepoints (Figure 7A). In contrast, Cluster 3 with 6 IR events show high PIR values during S-M timepoints and low PIR values during Light G1 and Dark G1 timepoints (Figure 7B). The gene expression data for the genes in Cluster 3 and 8 show that the mean expression fold change for these genes does not change significantly (< 2 fold) throughout the cell cycle (Figure 7C and 7D). The standard error at each timepoint is high due to low number of total IR events in each cluster. This suggests that most of these genes do not show large changes in gene expression and that IR is regulated independently of gene expression (Table 2 and Figure 8).

### PIR Cluster 3 and 8 events and their effects on the transcripts

Cluster 3 and Cluster 8 show high IR during the S-M and Dark G1 phase, respectively. To investigate the potential role of these genes and the impact of IR event on the coding potential of the transcript, we constructed gene – specific *de novo* transcripts from RNA-seq data using Trinity (Haas *et al.* 2013) (See Methods) and asked whether the IR event affects a known domain or introduces a PTC into the transcript. Of the eight genes in Cluster 8, seven genes have an annotated Pfam domain (summarized in Table 2 and Figure 8). IR in five of the seven genes introduces a PTC in the transcript. These events are depicted in Supplementary Figure S6. Interestingly, introduction of PTC in three genes is concordant with the low transcript level of these genes during dark phase. In the other two genes, the RPKM level of genes is not correlated with the PTC-introducing AS event.

**Table 2:** Intron retention Cluster 8 genes

Gene name	PfamID	Pfam Description	Effect of IR on annotated domain
Cre03.g152900 (MPA5)	PF00149	Calcineurin like phosphoesterase	Inserts 72aa in the cAMP domain
Cre08.g367650 (MUT11)	PF00400	WD40 Repeat domain	Introduce PTC in the transcript
Cre09.g395436 (RBM44)	PF00076	RRM domain	Introduce PTC in the transcript
Cre10.g456000	PF03358	NADPH – dependent FMN reductase	Domain not affected
Cre13.g574250	PF07714	Protein tyrosine kinase	Introduce PTC in the transcript
Cre16.g650800 (TIM13)	PF02953	Zinc-finger TIM10/DDP	Introduce PTC in the transcript
Cre16.g657979	—	—	—
Cre17.g741850 (HNR1)	PF14259	RRM domain	Introduce PTC in the transcript

IR events in Cluster 8 introduce PTC in two RRM – domain containing transcript *viz*. *HNR1* gene (Cre17.g741850) and *RBM44* gene (Cre09.g395436). *HNR1* gene encodes for a Heterogeneous Ribonucleoprotein (hnRNP) F/H protein. hnRNPs are versatile group of proteins that regulates pre-mRNA splicing, mRNA transport and translation (Geuens *et al.* 2016). Dominguez *Et Al*., (2010) showed that hnRNP F/H regulates splicing by sequestering G-tract positions in RNA that tend to form G-quadruplexes structure. This sequestration close to 5′ or 3′ splice sites and potentially promotes the recognition of RNA molecules by other spliceosomal components and thus regulates splicing (Dominguez *et al.* 2010). In this study, we found that first intron in the *HNR1* gene in *C. reinhardtii* is differentially retained during the cell cycle. This IR event introduces PTC in the *HNR1* transcript and disrupts the first RRM domain. During the Light G1 and early S-M phase (TP 1 to TP12.5), the PTC-introducing IR event shows low PIR value (0.0 to 0.030). But at TP13, the PIR value starts increasing (PIR: 0.388) and continues to rise till TP24 (PIR: 0.631). This suggests that the HNR1 protein is potentially active during the Light G1 and S-M phase and its transcript levels are regulated by IR during the diurnal cell cycle. This observation is concordant with the high transcript level of this gene during the light phase and low levels in the dark phase.

*RBM44* gene (Cre09.g395436), on the other hand, is annotated as subunit 4 in the splicing factor 3b (SF3b). The IR event in this gene also introduces PTC in the transcript, however there is no significant change in the transcript level during the cell cycle. SF3b complex is part of the core complex of the spliceosome machinery. Mutations in SF3b complex genes result into aberrant splicing and are linked to cancer (Cretu *et al.* 2016). EggNOG ortholog group mapping suggests that there are 16 homologs of SF3B subunit 4 in *Chlamydomonas* genome (EggNOG ID: KOG0131) but there is low sequence identity (< 23%) shared between these sequences and RBM44 protein sequence (Cre09.g395436). Also, the CLiP mutation profile shows that there are four insertions in the intronic region of this gene. There is likelihood of functional redundancy of SF3 subunit 4 gene in *Chlamydomonas* genome and therefore, the *RBM44* gene may not be essential. However, further work is needed to confirm this functional redundancy.

In Cluster 3, four out of six protein-encoding genes consist an annotated Pfam domain. IR in two of the four genes introduces PTC, however, we did not observe any significant change in the transcript levels of these genes (Table 3 and Figure 8). In two PTC introducing events, the IR occurs in the last intron of the gene, and thus, it is likely that these events are not affected by NMD (Lloyd 2018). Cre12.g514100 encodes for a protein kinase and is annotated as microtubule-associated kinase. IR in this gene introduce PTC in the transcript. The PTC-introducing transcript peaks during the dark timepoints, where there is change from PIR value of 0.1 at TP12 to 0.5652 at TP12.5 when cells transition from light to dark phase. However, there is no significant change in the RPKM level of the transcript during the cell cycle.

**Table 3:** Information on intron retention in Cluster 3 genes

Gene name	PfamID	Pfam Description	Effect of IR on annotated domain
Cre06.g248850 (PRPF4B)	PF07714	Protein Tyrosine domain	Last intron; introduce PTC in the transcript
Cre08.g370450 (MGE1)	PF01025	GrpE domain	Last intron; Does not affect the GrpE domain
Cre08.g386100	—	—	—
Cre10.g426632	—	—	—
Cre12.g514100	PF00069	Protein kinase domain	Introduce PTC in the transcript
Cre17.g704850	PF00156	Phosphoribosyl transferase domain	Last intron; introduce PTC in the transcript

*C. reinhardtii* is a model organism used to study many biological processes (Merchant *et al.* 2007). *Chlamydomonas* gene expression has been analyzed by microarrays, EST sequencing and RNA-seq under different conditions to capture target genes and gain new insights (Albee *et al.* 2013; Lv *et al.* 2013; Goodenough *et al.* 2014; Park *et al.* 2015; Tulin and Cross 2015; Zones *et al.* 2015; Wang *et al.* 2016). The RNA-seq data in most previous studies produced short reads (36 – 50 bp reads) that are difficult to use for analyzing AS events. In this study, we used time resolved RNA-seq data of *C. reinhardtii* (Zones *et al.* 2015) (100 bp paired end reads) to analyze AS events during the diurnal cell cycle. The analysis of longitudinal RNA-seq data of *C. reinhardtii* revealed 5371 AS events in 3278 genes (19.75% of a total of 17,706 genes), after applying stringent filters on PSI and PIR values and read counts associated with the AS event. Among these events, IR constitutes about 40% of the total AS events. These numbers are in concordance with the previous analysis of Raj-Kumar and colleagues who inferred AS events using collections of EST data from different conditions. The clustering of PSI values of alternative SJ revealed distinct patterns of alternative splice site usage during the cell cycle.

This study provides a high confidence set of AS events in *Chlamydomonas reinhardtii* that can be utilized in future studies to understand the transcript level regulation. The analysis also revealed a small set of AS events that show changes in the splicing pattern at different phases of the cell cycle. Further work will be needed to determine if these splicing events are functionally important for the *Chlamydomonas* cell cycle progression. A combined analysis of this data with *Chlamydomonas* mutants that affects splicing or NMD can help in further dissecting the post-transcriptional regulation in *Chlamydomonas* cells during the diurnal cell cycle.
